# Single-cell time series analysis reveals the dynamics of HSPC response to inflammation

**DOI:** 10.26508/lsa.202302309

**Published:** 2023-12-18

**Authors:** Brigitte J Bouman, Yasmin Demerdash, Shubhankar Sood, Florian Grünschläger, Franziska Pilz, Abdul R Itani, Andrea Kuck, Valérie Marot-Lassauzaie, Simon Haas, Laleh Haghverdi, Marieke AG Essers

**Affiliations:** 1 Berlin Institute for Medical Systems Biology, Max Delbrück Center in the Helmholtz Association, Berlin, Germany; 2 Institute for Biology, Humboldt-Universität zu Berlin, Berlin, Germany; 3 Division Inflammatory Stress in Stem Cells, German Cancer Research Center (DKFZ), Heidelberg, Germany; 4 Heidelberg Institute for Stem Cell Technology and Experimental Medicine (HI-STEM gGMBH), Heidelberg, Germany; 5 Faculty of Biosciences, University of Heidelberg, Heidelberg, Germany; 6 Division of Stem Cells and Cancer, Deutsches Krebsforschungszentrum (DKFZ) and DKFZ–ZMBH Alliance, Heidelberg, Germany; 7 Department of Hematology, Oncology and Cancer Immunology, Campus Benjamin Franklin, Charité - Universitätsmedizin Berlin, Corporate Member of Freie Universität Berlin and Humboldt-Universität zu Berlin, Berlin, Germany; 8 German Cancer Consortium (DKTK), Heidelberg, Germany; 9 Berlin Institute of Health (BIH) at Charité – Universitätsmedizin Berlin, Berlin, Germany; 10 Berlin Institute for Medical Systems Biology, Max Delbrück Center for Molecular Medicine in the Helmholtz Association, Berlin, Germany; 11 Charité-Universitätsmedizin, Berlin, Germany; 12 DKFZ-ZMBH Alliance, Heidelberg, Germany

## Abstract

Hematopoietic stem and progenitor cells (HSPCs) are known to respond to acute inflammation; however, little is understood about the dynamics and heterogeneity of these stress responses in HSPCs. Here, we performed single-cell sequencing during the sensing, response, and recovery phases of the inflammatory response of HSPCs to treatment (a total of 10,046 cells from four time points spanning the first 72 h of response) with the pro-inflammatory cytokine IFNα to investigate the HSPCs’ dynamic changes during acute inflammation. We developed the essential novel computational approaches to process and analyze the resulting single-cell time series dataset. This includes an unbiased cell type annotation and abundance analysis post inflammation, tools for identification of global and cell type-specific responding genes, and a semi-supervised linear regression approach for response pseudotime reconstruction. We discovered a variety of different gene responses of the HSPCs to the treatment. Interestingly, we were able to associate a global reduced myeloid differentiation program and a locally enhanced pyroptosis activity with reduced myeloid progenitor and differentiated cells after IFNα treatment. Altogether, the single-cell time series analyses have allowed us to unbiasedly study the heterogeneous and dynamic impact of IFNα on the HSPCs.

## Abstract (precis)

Unbiased single-cell time series analysis of gene expression response to inflammation, using novel computational approaches, links myeloid depletion to alterations in differentiation bias and pyroptosis activity.

## Introduction

Inflammation is the body’s evolutionarily selected immune response to infection or tissue damage. It not only results in the activation and consumption of immune cells but is also accompanied by significant alterations in the function and output of hematopoietic stem and progenitor cells (HSPCs). Identifying how inflammatory stress regulates the fate of HSPCs and affects their function has become the subject of thorough scientific investigation in recent years (Caiado et al, 2021). This started with our work and the work of others showing that pro-inflammatory cytokines such as IFNs (Essers et al, 2009; Baldridge et al, 2010), TNFα (Pronk et al, 2011) or IL-1 (Pietras et al, 2016) are able to induce proliferation of normally quiescent hematopoietic stem cells (HSCs). Further investigations on, for example, the mechanisms involved in stress-induced HSC activation or the response of progenitors have faced a significant challenge. Inflammation does not only impact the proliferation of hematopoietic cells, but it also induces extensive alterations in the expression of cell–surface proteins that are used as markers to distinguish different HSPC populations, with a strong increase in Sca-1 (Kanayama et al, 2020). This thus questions the reliability of using these surface markers in flow cytometry to identify and distinguish the different HSPC populations under inflammatory conditions. The recent development in single-cell expression profiling has advanced our understanding of HSPC heterogeneity (Watcham et al, 2019). Single-cell transcriptional profiles of HSPCs from these studies can now be used as reference datasets to identify individual HSPCs upon inflammation based on their transcriptional profiles, thus independent of cell–surface marker expression.

Whereas previous single-cell studies have examined the response of HSPCs to inflammation, these have been limited to single time points, providing only a snapshot of the response (Giladi et al, 2018). In contrast, we aimed to investigate the temporal dynamics of the HSPC compartment in response to inflammation, using a single-cell time series that spans the first 72 h of the acute inflammatory response in vivo. Analyzing such time series data is challenging because of the added temporal dimension and the recovering nature of the studied process. In addition, there are currently limited computational tools or pipelines available for processing, analyzing, and visualizing single-cell response time series data. To address this, we developed several novel computational approaches including unbiased cell type annotation for poststimulation time points, characterization of the change in gene expression in each cluster across time points, and a semi-supervised (i.e., using the cells’ actual time labels as input) solution to recover the gene expression dynamics over response pseudotime. The set of methods we used in these analyses is bundled in a computational pipeline that helped us identify global and cluster-specific gene expression dynamics associated with different biological responses in HSPCs after IFNα treatment, with HSCs being the main and strongest responders to IFNα. Importantly, we also uncovered a reduction of myeloid progenitor cells associated with changes in transcriptional programs in multiple clusters. Thus, our single-cell time series analyses have helped us better understand how different cell types, genes, and processes change, whereas the HSPC compartment progresses through the inflammatory response. In addition, our novel pipeline designed for posttreatment single-cell time series data will be a useful tool for future analysis of response time series datasets.

## Results

### A single-cell time series dataset capturing the dynamic inflammatory response of HPSCs

**Figure 1.**
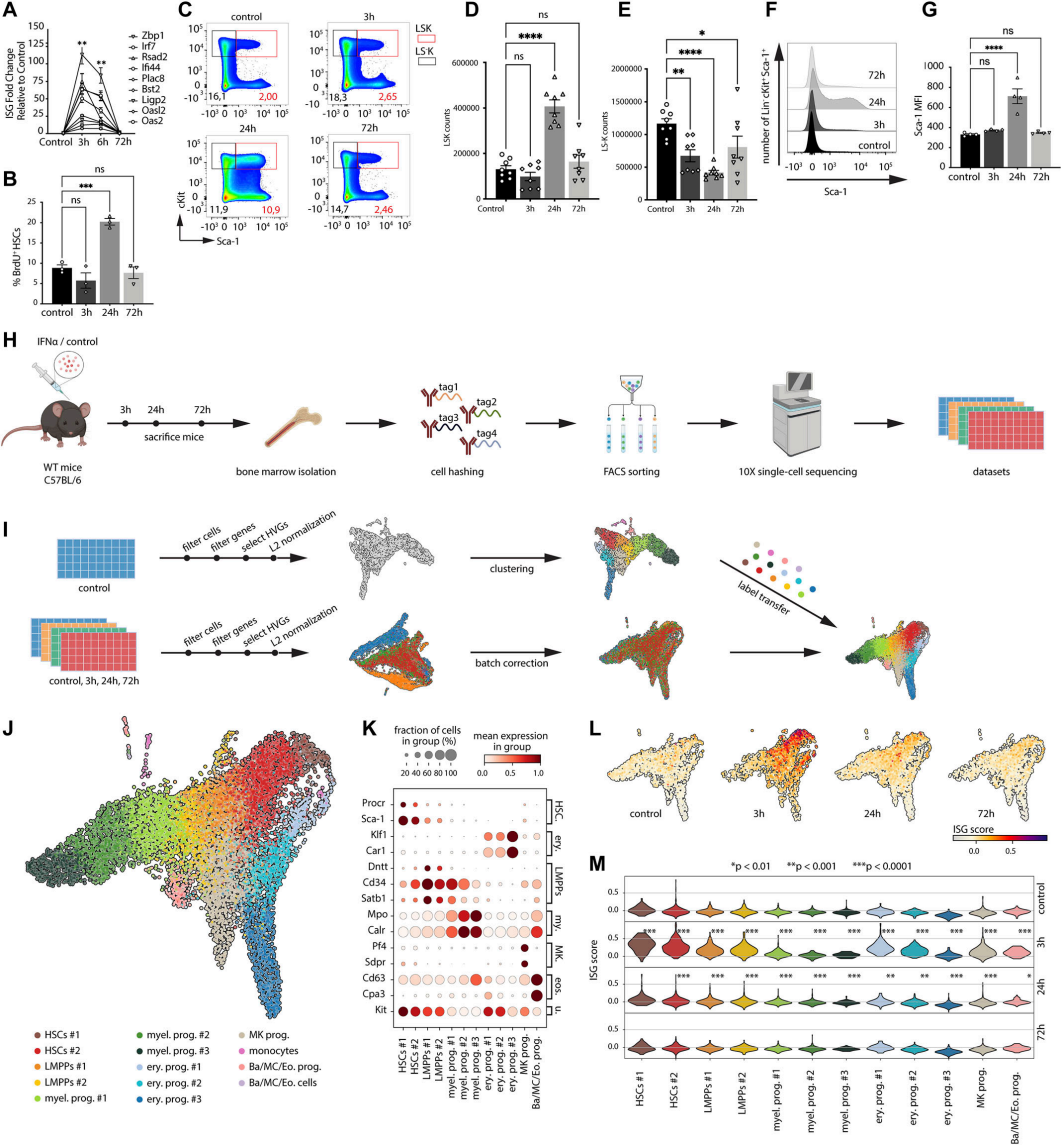
A single-cell time series RNA-seq dataset to characterize the response of HSPCs to IFNα treatment. **(A)** Gene expression levels of interferon-response genes in HSCs (Lin^−^ Sca-1^+^ cKit^+^ CD150^+^ CD48^−^ CD34^−^) from control (PBS) or IFNα-treated WT mice (50,000 IU/20*g* mouse; 3, 6, and 72 h) were quantified using qPCR. Three biological replicates were used in the analysis. **(B)** Cell proliferation measured by 14 h BrdU (18 mg/kg) uptake of HSCs (Lin^−^ Sca-1^+^ cKit^+^ CD150^+^ CD48^−^ CD34^−^) from control (PBS) or IFNα-treated WT mice (50,000 IU/20*g* mouse; 3, 24, and 72 h). n = 3 biological replicates. **(C)** Representative FACS plots of Sca-1 and cKit expression on Lin^−^ BM cells after control or 3, 24, and 72 h time course IFN⍺ treatment. **(D, E)** Cell counts of Lin^−^ Sca-1^+^ cKit^+^ (LSK) (D) and Lin^−^ Sca-1^−^ cKit^+^(LS^−^K) (E) cells in BM after control or 3, 24, and 72 h time course IFN-α treatment. n = 8 biological replicates. **(F, G)** quantification (F) and statistical analysis (G) of Sca-1 median fluorescence intensity on Lin^−^ cKit^+^ cells. **(H)** Scheme illustrating the experimental steps to acquire the single-cell time series RNA-sequencing dataset. **(I)** Scheme of the computational pipeline used to process the time series dataset. **(J)** Two-dimensional UMAP embedding of cells from all time points, colored for the different identified clusters as indicated in the legend. **(K)** Expression of marker genes in the different clusters in the control dataset. Erythroid (Ery.); myeloid (My.); basophils, mast cells and eosinophils, (Ba/MC/Eo.); universal (U.). **(L, M)** UMAP embeddings (L) and violin plot (M) of the interferon response gene score (see the Materials and Methods section) in the different clusters in the four different time points. Statistical significance in (A, B, D, E, G) was determined by an ordinary one-way ANOVA using Holm-Šídák’s multiple comparisons test. **(A, D, E, G)** At least three independent experiments were performed (data in (A) shows a representative experiment, data in (D, E) are representative of two independent experiments, and data in (G) shows a representative experiment); *P ≤ 0.05, ***P ≤ 0.001, ****P < 0.0001. Statistical significance in (L) was determined by a one-sided Wilcoxon rank-sum test between control and each treatment time point for each cluster.

Biological responses such as acute inflammation are dynamic processes in which cells, tissues, and organisms undergo different phases of sensing differences, responding to these changes, and recovering upon successful response. Yet, often only single time points of these responses are investigated. Quiescent HSCs respond to inflammation by increased proliferation, which can be mimicked by treating mice with single pro-inflammatory cytokines, such as IFNα. To gain a better understanding of the dynamics of the HSCs response to acute inflammation, we performed a time series experiment to cover the sensing, response, and recovery phases of the inflammatory response of HSCs to treatment with IFNα. Whereas at 3 h post-treatment, HSCs showed the first signs of sensing IFNα by increasing expression of interferon-stimulated genes (ISGs) (Fig 1A), only at 24 h posttreatment, HSCs reached a peak in increased proliferation (Fig 1B) (Essers et al, 2009; Pietras et al, 2014). 72 h posttreatment, ISG expression was back to baseline (Fig 1A) and most of the HSCs returned to quiescence (Fig 1B). Unfortunately, inflammation does not only lead to increased proliferation of HSCs but is also accompanied by increased expression of several cell surface protein markers used to identify different cell types within the HSPC compartment. The most well-known example is the increase in the stem cell marker Sca-1 (Essers et al, 2009; Pietras et al, 2014; Kanayama et al, 2020). Using conventional marker-based flow cytometry including Sca-1, an increase in the more stem-like LSK (Lin^−^ Sca-1^+^ cKit^+^) cells and a decrease in the more committed LS^−^K (Lin^−^ Sca-1^−^ cKit^+^) progenitors is observed in response to inflammation (Fig 1C–E). However, because of the increase in protein expression of Sca-1 (Fig 1F and G) it is hard to predict whether these changes in frequency reflect an actual increase/decrease in cell frequency or are the result of contamination because of changes in protein expression of the cell markers in a given population or even both.

**Figure S1.**
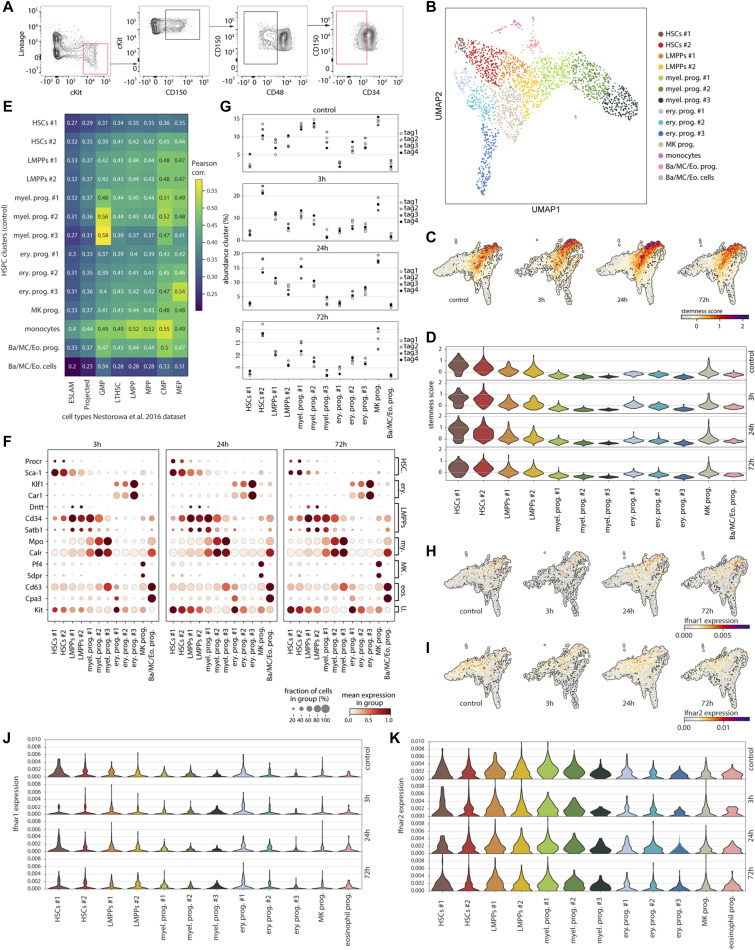
Characterization of cell clusters in the single-cell HSPC dataset. **(A)** Gating strategy for the single-cell RNA-sequencing experiment, where 10,000 Lin^−^ cKit^+^ cells were sorted and enriched with 3,000–5,000 HSCs (Lin^−^ cKit^+^ CD150^+^ CD48^−^ CD34^−^). Cells sorted are within the red gates. **(B)** Two-dimensional UMAP embedding of cells from the control subset, colored for the different identified clusters as indicated in the legend. **(C, D)** UMAP projection (C) and violin plot (D) of stemness score (see the Materials and Methods section) in the IFN⍺-treated or control (PBS) subsets. **(E)** Pearson correlation between gene expression of clusters in our HSPC dataset versus the cell types in the HSPC dataset from Nestorowa et al (2016). **(F)** Expression of marker genes in the different clusters in the 3, 24, and 72 h IFN⍺-treated subsets. erythroid (ery.); myeloid (my.); eosinophils (eos.); universal (U.). **(G)** Relative abundances of each cluster for the four different hashtags (biological replicates) in each timepoint. **(H, I)** UMAP projection of *Ifnar1* (H) and *Ifnar2* (I) expression in the four subsets. **(J, K)** Violin plot of *Ifnar1* (J) and *Ifnar2* (K) expression in the four subsets.

To overcome these limitations, we adopted a single-cell RNA sequencing (RNA-seq) approach to investigate the dynamics and heterogeneity of the stress response of stem and progenitor cells to IFNα treatment. Bone marrow cells were collected from IFNα-treated mice 3, 24, and 72 h after treatment. Cells from PBS-treated mice were included as a control. LK (Lin^−^ and cKit^+^) cells were sorted to capture a wide spectrum of the HSPC transcriptional landscape. Because HSCs are much less frequent than the other populations in the LK gate, we enriched the sorted LK samples with LK CD150^+^ CD48^−^ CD34^−^ cells at a fixed ratio to the number of LK cells to guarantee sufficient numbers of stem cells for analysis (Fig S1A). Inter-animal heterogeneity of the inflammatory stress response was addressed by performing cell hashing, for which cells from each biological replicate and time point were labeled with a unique hashtag antibody (Fig 1H and the Materials and Methods section). The cells from all four experimental time points (control, 3, 24, and 72 h) were sequenced simultaneously. The resulting dataset was processed using a computational pipeline that we designed specifically for poststimulation single-cell time series (Fig 1I). First, the cells that did not meet the quality control standards (e.g., doublets, dying cells) were removed, resulting in a total count of 1,600–3,500 cells per time point (see the Materials and Methods section). Next, clustering of the cells identified 14 different clusters in the control subset (Fig S1B). Using known marker genes for HSPC types, a scoring for stemness and a comparison with a previously published dataset (Nestorowa et al, 2016), eight different cell types could be distinguished in the control subset (Figs 1J and K and S1C–E). To compare the identified cell with to their equivalents in the treatment subsets, the cell type labels were transferred from the control subset to the other three treatment time points (Fig 1I and the Materials and Methods section). To do this without the treatment affecting cell type labels, data from all four time points were “batch-corrected.” In the batch-corrected datasets, cells in posttreatment timepoints were labeled based on their nearest neighbors in the control dataset. In addition, the batch-corrected data were used to produce a UMAP where cells are located based on their cell type identity, rather than their experimental timepoint (see difference between the two bottom UMAPs in Fig 1I). It is important to point out that the batch-corrected data were only used for label transfer and visualization purposes, but for all downstream analyses, the original data were used. Marker gene expression confirmed the cell type labels in the response time points (Fig S1F). Analysis of the hashtags showed that the biological replicates in each time point had comparable abundances of each cell type label (Fig S1G). In addition, expression analysis of the IFN α/β receptor (*Ifnar1* and *Ifnar2*) confirmed that all clusters expressed the IFN α/β receptor and thus were able to directly respond to IFNα (Fig S1H–K). As a first measure of the inflammatory response, the expression of ISGs was scored (Fig 1L and M). At 3 h, all clusters underwent a change in their ISG expression, indicating that the whole HSPC compartment sensed the IFNα treatment (Fig 1L). However, the data also indicate great heterogeneity in the ISG response between and within clusters with HSC clusters showing the biggest change (Fig 1M).

### Inflammation response is defined by global and cluster-specific changes in gene expression

**Figure 2.**
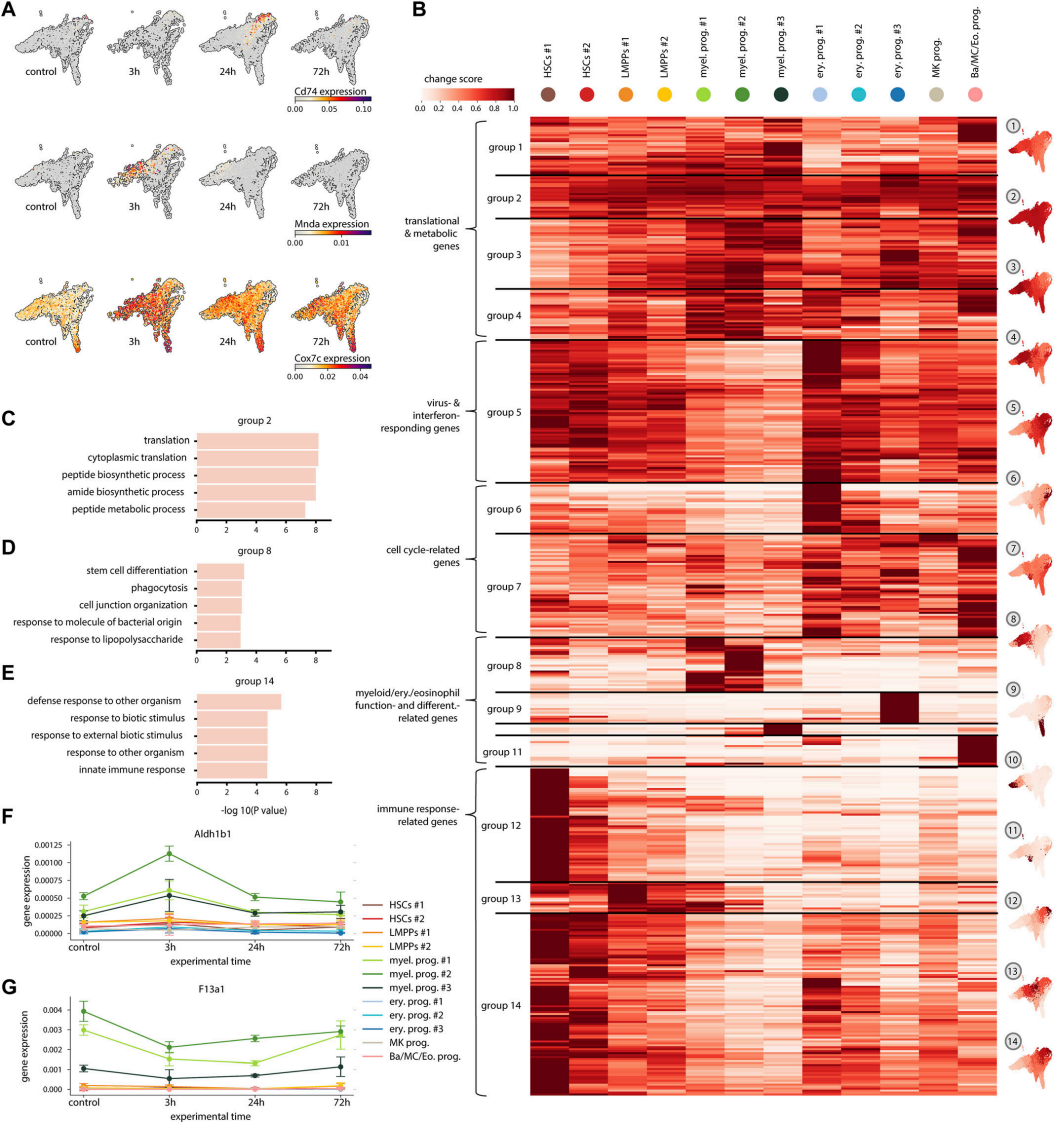
Inter-cluster analysis of response genes shows both global and cluster-specific responding genes. **(A)** UMAP embeddings with the expression of response genes *Cd74*, *Mnda*, and *Cox7c* in control and 3, 24, and 72 h post IFN⍺ treatment. **(B)** Change score (see the Materials and Methods section) in each cluster for the top 500 response genes, grouped using hierarchical clustering. UMAPs on the right show the expression change for each cell cluster averaged over all response genes in the corresponding group. On the left are terms summarizing the functional annotation of the response genes associated with the groups. **(C, D, E)** GO terms associated with groups 2 (C), 8 (D), and 14 (E). The length of each bar represents the statistical significance of each term. **(F, G)** Mean expression of *Aldh1b1* (F), and *F13a1* (G) in each cluster over time.

**Figure S2.**
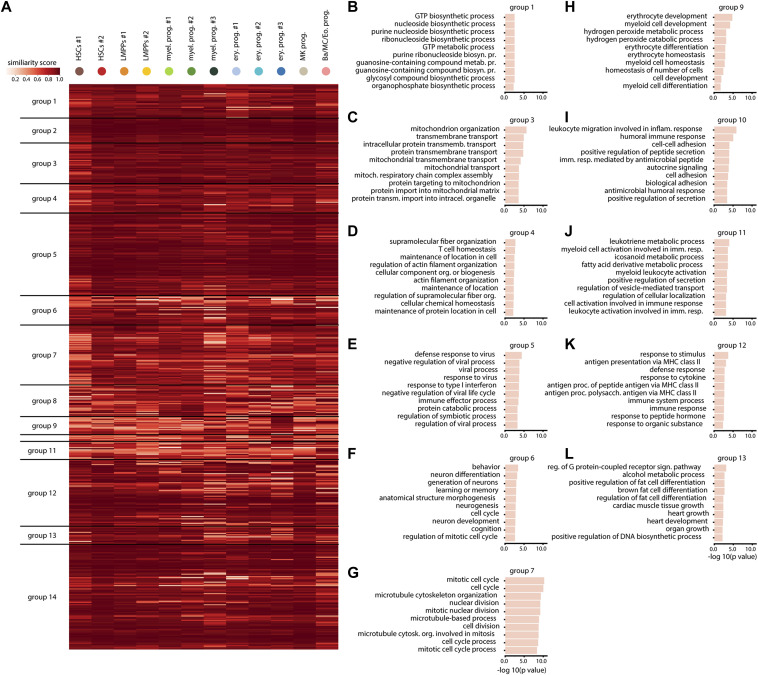
Inter-cluster similarity score analysis confirms the validity of cluster-specific inflammation signatures. **(A)** Similarity score (between 0 and 1) illustrating the similarity between the pattern of a gene in a specific cluster and the average pattern in the whole dataset. Genes are grouped as in Fig 3B. **(B, C, D, E, F, G, H, I, J, K, L)** GO terms significantly enriched in the different change score groups (as defined in Fig 3B). The length of each bar represents the statistical significance of each term.

To gain a comprehensive understanding of all genes that characterize the IFNα response, we next performed differential gene expression analysis. Differentially expressed genes (DEGs) were selected between the control subset and every treatment time point individually to get a set of response genes from any stage of the response (see the Materials and Methods section). The analysis identified a total of 2,501 significant response genes. Expression profiles of the response genes showed that the expression of some genes changed globally (e.g., *Cox7c*), whereas the expression of others was more specifically changed in few or one cluster (e.g., *Sec61g* and *Mnda*) (Fig 2A). To investigate in which cluster(s) genes were changing the most, the top 500 most significant response genes were scored for the total expression change in each cluster (change score; see the Materials and Methods section). After calculating the total change for each cluster, the response genes were categorized into 14 different groups using hierarchical clustering (Fig 2B), confirming a wide variety of globally responding genes (groups 1–5), and cluster-specific responding genes (groups 6–14). To exclude that the differences between clusters were a result of completely different expression profiles, the similarity between a cluster’s expression profile and the expression profile in the whole dataset was calculated (Fig S2A). The results indicate that most expression profiles follow similar patterns in all clusters.

Gene ontology (GO) enrichment analysis of the global response gene signatures in groups 1–4 revealed an overrepresentation of terms associated with translation and metabolism (Figs 2C–E and S2B–D). This is in line with reports of HSPCs undergoing massive changes in the metabolism under inflammatory stress (Karigane & Takubo, 2017). In addition, global response genes from group 5 (Fig S2E) showed enrichment for categories associated with immune response and response to type-I IFN, further supporting the ISG expression data (Fig 1K), indicating that all cells sense the changes in IFNα levels. The GO enrichment terms for other gene groups are shown in Fig S2F–L. Interestingly, expression changes in the HSC-enriched groups 12 and 14 were also associated with immune response and response to type-I IFN (Figs 2E and S2K). However, these changes were different from the changes in group 5, suggesting an HSC-specific immune response, which is different from the immune response in progenitors. HSC-enriched groups 12 and 14 also included GO terms such as regulation of T cell activation and antigen processing and presentation (Figs 2E and S2K), which correspond to the newly identified role of HSCs as immunomodulators (Hernández-Malmierca et al, 2022), which would be strengthened under inflammation. Besides global and HSC-specific response genes related to immune response, change score analysis also identified groups of response genes enriched in committed progenitors related to progenitor-specific processes. For example, erythroid and basophil/mast cell/eosinophil progenitor-enriched groups 9 and 11 showed an overrepresentation of processes related to erythrocyte differentiation terms and myeloid development (Fig S2H and J). Change scores in groups 8 and 10 were largest for myeloid progenitors and connected with biological processes such as phagocytosis, myeloid leukocyte-mediated immunity, and stem cell differentiation, which are characteristic functions of this cell type (Figs 2D and S2I). Thus, with the change score analysis both global and cluster-specific signatures were identified. The analysis highlights that HSCs are the major responders to inflammation in the HSPC compartment and both global and HSC-specific inflammation signatures are present, indicating heterogeneity in the inflammatory response between the clusters.

### The pseudotemporal ordering of cells enhances the resolution of gene dynamics

**Figure 3.**
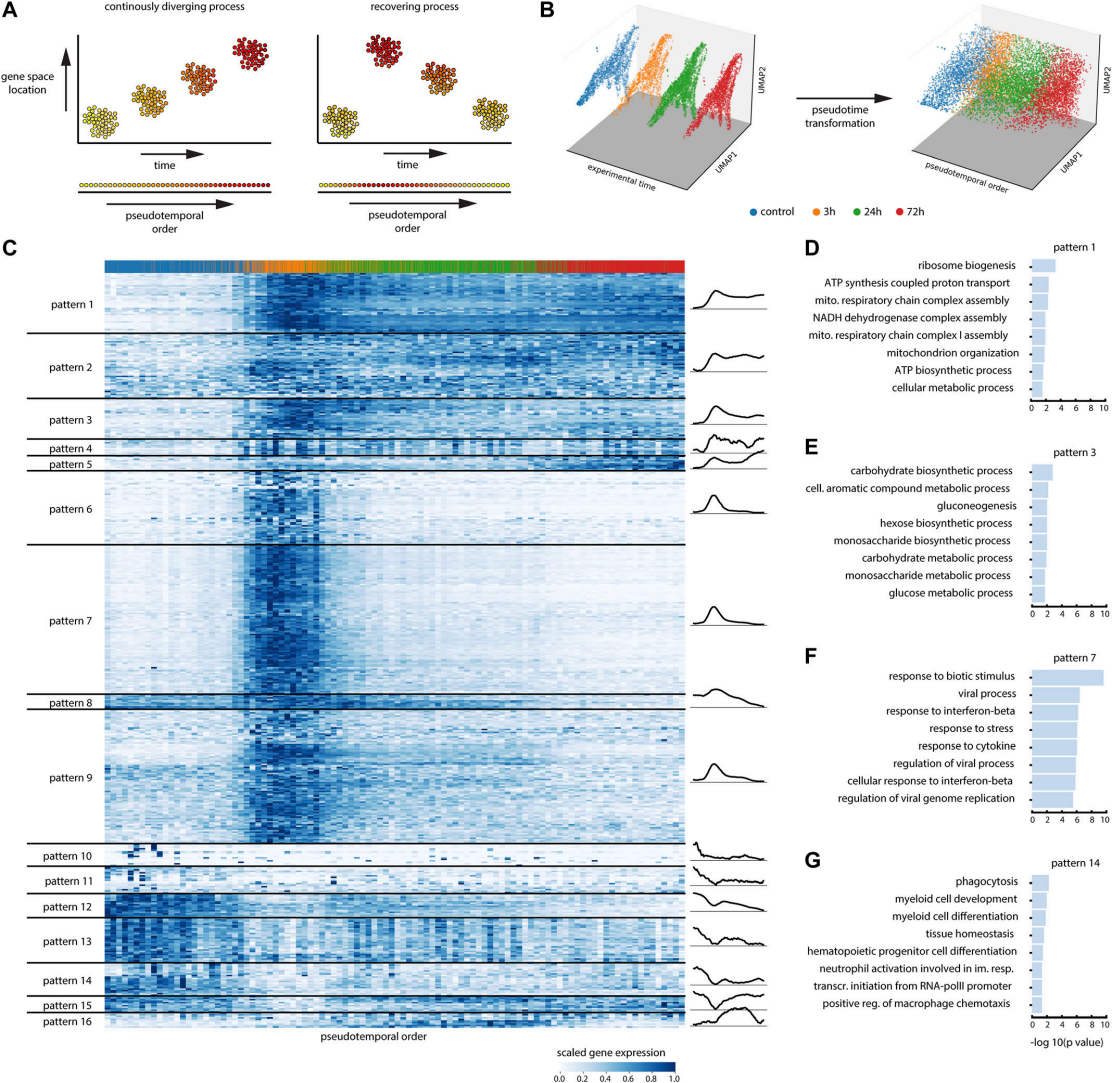
Implementing response pseudotime to characterize the dynamics of gene expression in response to IFN⍺ treatment. **(A)** Illustration of the difference between time series that capture continuously diverging processes (such as development), and recovering processes (such as acute stimulation). **(B)** Three-dimensional embedding of the HSPC dataset with experimental timepoints (left) or pseudotemporal ordering (right) on the z-axis (x- and y-axis: UMAP). **(C)** (smoothed) Expression of the top 500 response genes, with cells ordered by pseudotime and genes grouped by pattern using hierarchical clustering. A graphical representation of the mean pattern in each pattern group is shown on the right. **(D, E, F, G)** GO terms associated with gene patterns 1 (D), 3 (E), 7 (F), and 14 (G). The length of each bar represents the statistical significance of each term.

**Figure S3.**
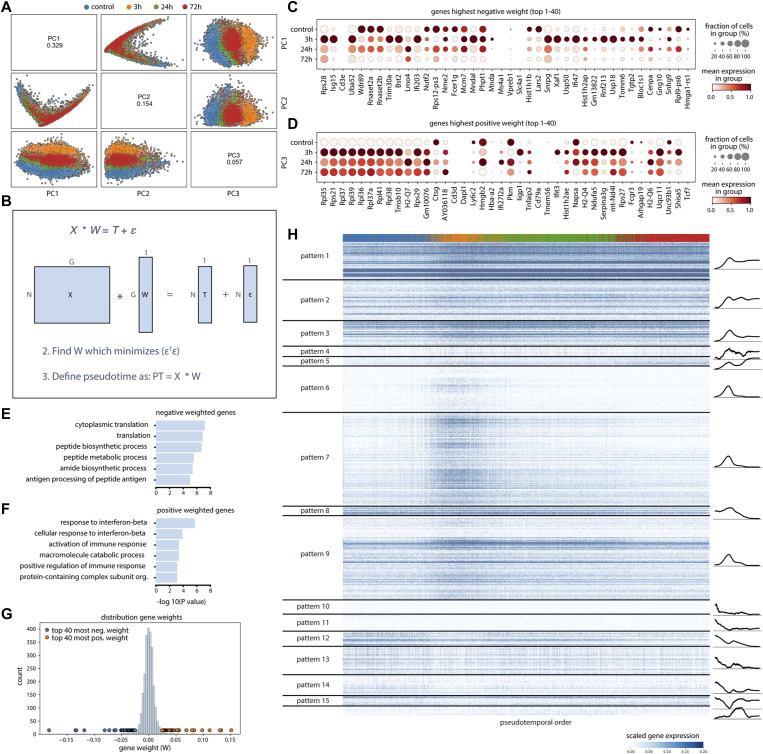
The response pseudotime ordering of cells. **(A)** Principal components 1–3 of the non-batch–corrected HSPC dataset. **(B)** The response pseudotime for each cell is calculated by solving a linear regression for the weight matrix W that transforms the data matrix X (with N cells and G genes) to the experimental time labels vector T, with minimal error (ε). **(C, D)** gene expression of the top 40 genes with the lowest negative association (i.e., weight in the W matrix) with pseudotime (C), gene expression of the top 40 genes with the highest positive association with pseudotime (D). **(E, F)** GO terms associated with negative weighted genes (E) and positive weighted genes (F). The length of each bar represents the statistical significance of each term. **(G)** All weights in vector W, represented as histogram with points representing the top 40 most positive weighted genes (orange) and top 40 most negative weighted genes (blue). **(H)** Non-smoothed expression of the top 500 DEGs. Expression patterns were grouped as in Fig 3C.

**Figure S4.**
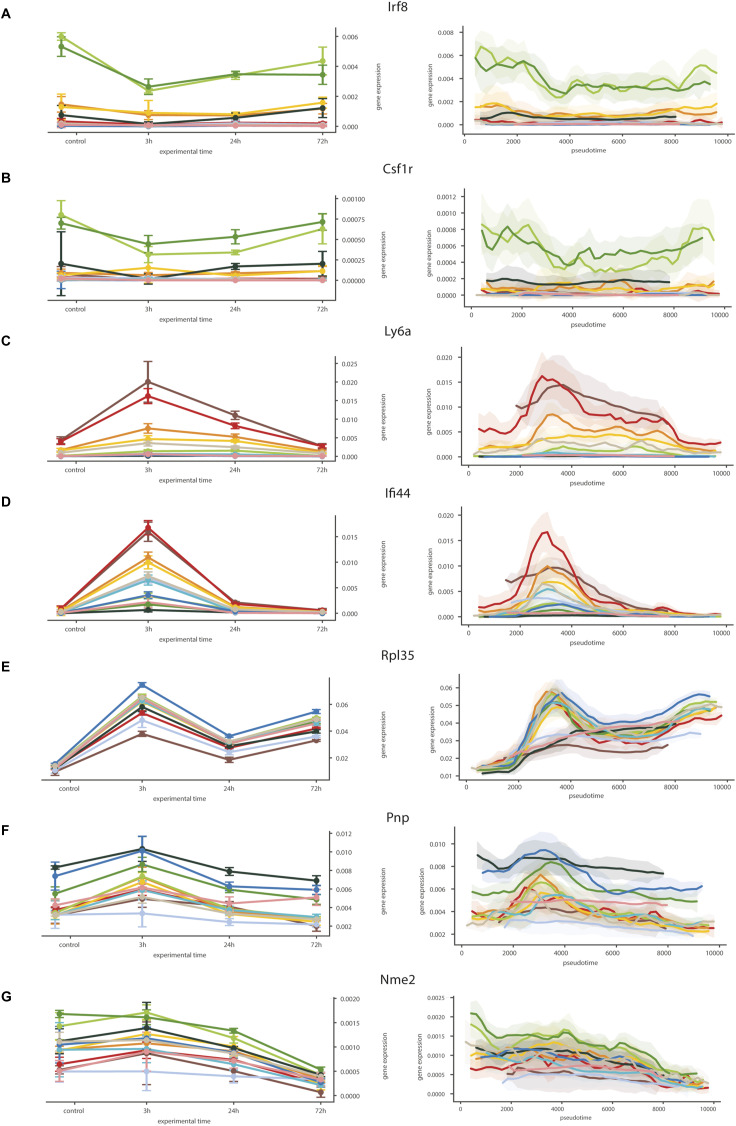
Validation of pseudotime gene expression dynamics. **(A, B, C, D, E, F, G)** Actual time series dynamics represented as mean discrete expression for each of the clusters (right) versus the reconstructed pseudotemporal expression in the different clusters (left) for the same genes *Irf8* (A), *Csf1r* (B), *Ly6a* (C), *Ifi44* (D), *Rpl35* (E), *Pnp* (F), *Nme2* (G).

**Figure S5.**
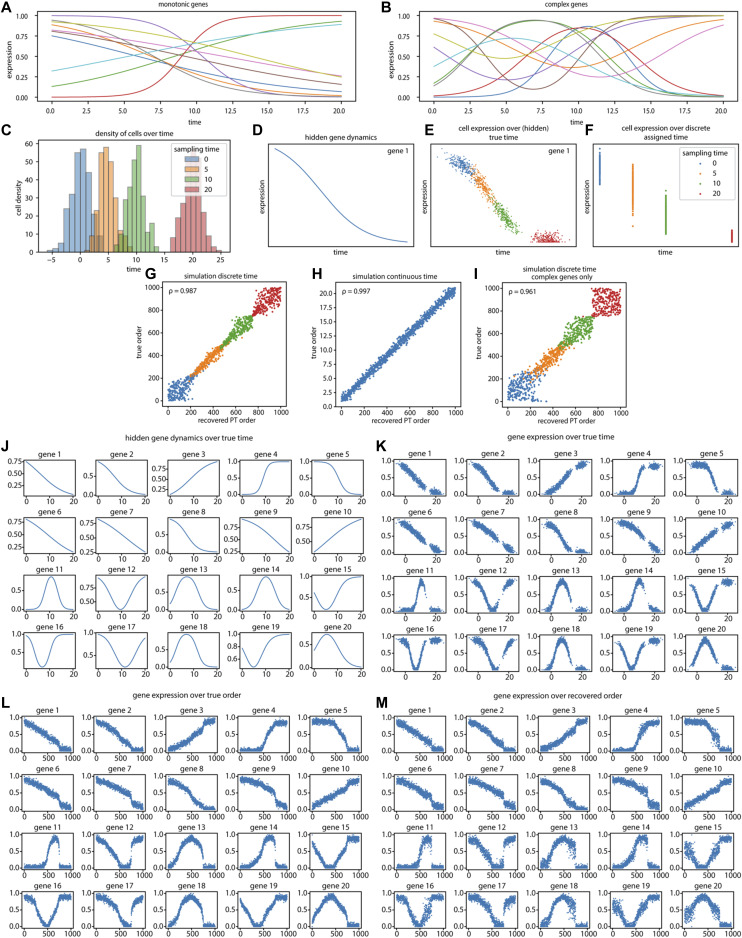
Response pseudotime recovers gene expression dynamics in simulation. To test the performance of our response pseudotime approach, we applied our method to a simulation. **(A)** The simulation included the temporal dynamics of a total of 20 stimulated genes that fall into two categories: (A) monotonic genes that mimic genes that are up-regulated or down-regulated without recovery within the covered time interval. **(B)** Complex genes that are up- or down-regulated but also demonstrate a form of recovery within the timeframe of the simulation. **(C)** After adding Gaussian noise—one for modeling the biological variance in gene expression and one for modeling the asynchronous progression of cells through time—1,000 cells were sampled from four different timepoints. This figure shows the density of cells in each of the four simulated capture (experiment) timepoints across the (continuous) true simulation time values. **(D, E, F)** Each gene in the simulation has hidden gene dynamics, which theoretically explain the gene expression over true time (D). **(E)** In a simulation, we have access to the true time assignment of each cell (E). However, real-world single-cell time series datasets do not include a measure of true time for cells. The only measurements of time that we can use are the experimental timepoints. **(F)** Therefore, the data in our simulation are transformed to mimic the discrete experimental timepoints (F). **(G)** We used our response pseudotime to recover the temporal order of the cells in the dataset. In (G), we compare the recovered response pseudotemporal (PT) order of the cells with the true temporal order of cells. To describe the accuracy of the recovery we use a Spearman’s correlation test (ρ), which demonstrates that we are able to recover the true temporal order with a high accuracy. **(H, I)** In addition, we also tested our response pseudotime method on a simulation where the cells are sampled uniformly along the true time axis (H) and a simulation which included only complex genes (I). The latter illustrates that our approach even has reasonable accuracy in scenarios where all genes (partially) recover during the measured time frame. **(J, K, L, M)** To illustrate how the gene dynamics are recovered using our response pseudotime approach, we visualized the hidden dynamics of all genes in the simulation (J), and plotted the gene expression of the sampled cells over true time (K), true order (L), and recovered PT order (M). **(J, K, L, M)** The reader might notice that the gene dynamics in (L, M) look slightly warped in comparison with the dynamics in (J, K). This is an unavoidable consequence of a time series dataset if the cells’ sampling function along true time is unknown (non-uniform, non-stationary state, etc.). Consequently, when we order the cells, there is more coverage of certain parts of the true time than others which will warp the gene dynamics.

The change score analysis gave an overview of all response genes without taking into account the detailed dynamics of the response. Therefore, in the next step, the expression dynamics of the response genes were explored. When zooming into the expression of individual genes in time, different expression patterns were observed for genes within the same group. For example, the responses of *Aldh1b1* and *F13a1* were both assigned to group 8. However, the temporal expression dynamics differed between those genes; whereas *Aldh1b1* was up-regulated with a peak at 3 h and a full recovery to original (control) expression levels at 24 h (Fig 2F), *F13a1* expression steadily went down (Fig 2G). To improve the characterization of the expression patterns, we wanted to leverage the single-cell resolution of our dataset. Therefore, we aimed to construct a pseudotime axis in the gene expression space to describe the inflammatory response. In datasets covering a developmental process or disease progression, the asynchrony of cells can be leveraged to infer a pseudotemporal ordering of cells (using methods such as diffusion pseudotime [Haghverdi et al, 2016], Monocle [Qiu et al, 2017], etc.). These methods are generally based on cell neighborhood relations, with the assumption that the further apart (in Euclidean, diffusion space, etc.) two cell states are, the longer the typical transition time between them, hence longer pseudotime (Fig 3A). However, this assumption is violated for the type of post-drug treatment time series data we have here, where the largest transcriptional change is observed shortly after stimulation but (presumably) diminishes as cells relax to a more control-like state over a longer time (Fig 3A). In our datasets, cells from different experimental time points generally appear either completely intermingled or completely disconnected from one another when viewed over the first principal components (Fig S3A). This renders the unsupervised, distance-based pseudotime methods unsuitable for detecting the temporal order of response dynamics. Thus, to get a higher temporal resolution of the response progression from the four time points, we used a semi-supervised (using the experimental time labels) approach that finds a pseudotime axis that best correlates with the cells experimental time labels. We find a transformation of the cells’ expression matrix that reconstructs the experimental time labels plus an error term that we try to minimize (Fig S3B and the Materials and Methods section). In essence, this approach assigns a positive/negative weight to each gene (depending on its correlation or anticorrelation_across all cell types_ with the experimental time labels), in such a way that the resulting weighted sum of the gene expression profile of a cell approximates its experimental time labels up to an error term, which we interpret as the asynchrony of treatment response among different cells captured at the same experimental time point. Using this approach implies that there are at least a few genes in the data, a linear combination of which can be used to differentiate the different time points. Interestingly, the genes given highest positive weights by the model turn out to be often among the global changing ones, and associated with the immune response, whereas the genes assigned negative weights display enrichment in processes such as translation (Fig S3C–G). In Fig S3H, we see the non-smoothed counterpart, thus sparser expression matrix of the same genes as that of Fig S3C, which confirms the robustness of the inferred patterns. We further confirm the agreement of actual time series dynamics with that of the reconstructed pseudotime pattern for a few show-case genes (Fig S4A–G). In addition, we introduce a simulation dataset on which we demonstrate the properties of the inferred pseudotime order, and use it for evaluation of the method’s performance (the Materials and Methods section and Fig S5A–M).

Using the response pseudotemporal order of the cells, we moved from the four time points’ discreet view of the data to a continuous axis (Fig 3B) over which the different expression dynamic patterns that follow IFN⍺ treatment were explored. Response genes were categorized into 16 patterns based on their pseudotemporal dynamics using hierarchical clustering (Figs 3C and S3H). Each pattern represents a cluster of genes with similar expression dynamics after IFN⍺ injection, which can be roughly subdivided in up-regulation (patterns 1–9 and 16) and down-regulation (pattern 10–15) patterns. The GO enrichments for the gene patterns are shown in Fig 3D–G. The patterns showed a broad diversity in the speed of sensing, response, and recovery. Whereas most patterns reach a steady-state plateau towards the end of the pseudotime axis, a few (patterns 5, 8, and 12) do not, and imply an ongoing trend of changes beyond the covered 72 h posttreatment genes in patterns 6, 7, and 9 show quick sensing, response, and recovery associated with immune, IFN, and viral processes (Fig 3F). Other patterns resembled a similar fast increase but with slower recovery, as seen for pattern 3, which is enriched for metabolic processes (Fig 3E). In addition, the heatmap in Fig 3C showcases a variety of gene dynamics that were different from the rapid response and recovery IFN-response (patterns 6, 7, and 9), such as a sustained up-regulation in pattern 1, which was associated with translation and other biosynthetic processes (Fig 3D). In contrast, several gene patterns encompassed genes that were down-regulated (gene patterns 10–14). After the initial decrease in expression, many of these genes failed to recover to initial expression levels. Most of these genes were linked to myeloid development and differentiation (Fig 3G). This would suggest alterations in myeloid differentiation upon the IFN⍺ treatment, an observation described for many other pro-inflammatory cytokines (Matatall et al, 2014; Pietras et al, 2016; Yamashita & Passegué, 2019) but IFN⍺.

### Response pseudotime reveals a landscape of gene dynamics in HSPCs after IFN⍺ treatment

**Figure 4.**
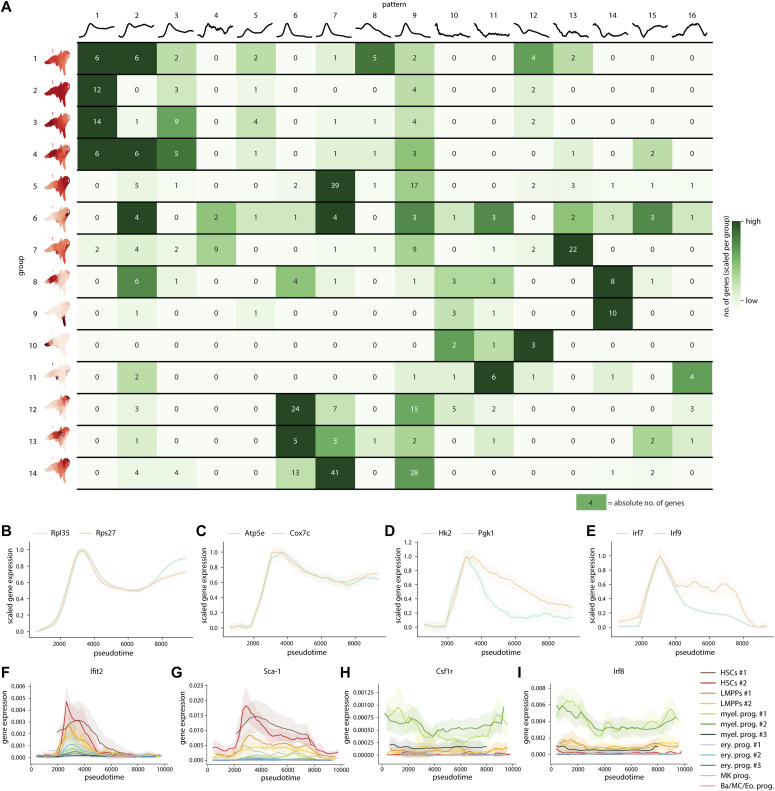
Response pseudotime reveals a landscape of gene dynamics in HSPCs following IFN⍺ treatment. **(A)** Visual summary of the HSPC time series showing the breakdown of the response gene patterns in each change score group. The numbers in each cell represent the absolute number of genes (e.g., five response genes in change score group 1 display pattern 1). The colors represent the number of genes scaled for each change score group. **(B, C, D, E)** Examples of gene expression in pseudotime for translation (*Rpl35*, *Rps27*) (B), metabolism (*Atp5e*, *Cox7c*, *Hk2*, *Pgk1*) (C, D), and inflammation (*Irf7*, *Irf9*) (E) specific genes. **(F, G, H, I)** Pseudotemporal expression of HSC-specific genes *Ifit2* (F) and *Sca-1* (G) and myeloid-specific genes *Csf1r* (H) and *Irf8* (I) in the different clusters.

To decipher the dynamic changes in the inflammatory response in the different clusters, we combined the information on how response genes changed their expression (Fig 3C) with whether these changes were global or cluster-specific (Fig 2B). The result condenses the plenitude of information in the complete single-cell time series into a single visualization, which considerably eases the search for (groups of) biologically relevant genes (Fig 4A).

The most commonly found patterns among the groups are patterns 1, 2, 7, and 9, showing a fast up-regulation combined with a partial (patterns 1 and 2) or full (patterns 7 and 9) recovery. Genes from these patterns are mainly related to immune responses, highlighting that the sensing and response to IFN⍺ is fast and present in all groups and clusters. The full recovery in the most common patterns 7 and 9 revealed by our single-cell experiment indicates that the general immune response does fully recover within 72 h, a fact that bulk experiments had not captured before.

Interestingly, other patterns of fast sensing and response followed by sustained up-regulation (patterns 1 and 3) were enriched in the groups with a global signature (groups 1, 2, 3, and 4). Several of these genes were ribosomal (e.g., *Rpl35* and *Rps27*; Fig 4B), suggesting that biosynthetic activity increased in most HSPCs early in the treatment and remained active even in the recovering phase, when proliferation levels are back to homeostasis again (72 h; Fig 1B). In addition, several genes after the sustained up-regulation pattern were metabolic. Oxidative phosphorylation (OXPHOS) genes and mitochondrial enzymes (e.g., *Atp5e* and *Cox7c*; Fig 4C) showed these patterns of prolonged up-regulation. On the other hand, glycolytic genes *Hk2* and *Pgk1* showed a quick response and recovery (Fig 4D). Thus, in contrast to previous reports suggesting a binary (on/off) switch between glycolysis and OXPHOS (Suda et al, 2011), our data suggest that an initial up-regulation of glycolytic and a sustained up-regulation of OXPHOS genes go hand in hand in inflammation responding HSPCs.

In contrast to the heterogeneity in dynamics observed globally, HSC-enriched groups (groups 5, 12, and 14) are mainly enriched with gene patterns that increase very early after treatment and quickly return to homeostatic levels (patterns 6, 7, and 9). Examples of such genes are *Irf7* and *Irf9* in group 5 (Fig 4E) or *Ifit2* and *Sca-1* in groups 12 and 14 (Fig 4F and G). Thus, most of the HSC-enriched groups follow rapid sensing, responding, and recovery dynamics, with most of the gene changes preceding the peak in proliferation response in these cells. In addition, most of the HSC-enriched dynamic changes are within gene groups linked to IFN and immune response, again highlighting the specific, fast HSC-specific immune response.

In contrast to HSC-specific groups, committed progenitor-specific groups (8, 9, 10, and 11) were strongly enriched in genes that exhibited persistent down-regulation (patterns 10, 11, 12, and 14). In the myeloid progenitor-specific groups 8 and 10, many of these genes were associated with myeloid cell differentiation and functional programs, e.g., Csf1r; Irf8 (Fig 4H and I), suggesting reduced myeloid differentiation in the myeloid progenitor clusters.

In summary, response pseudotime has shed light on the heterogeneity in gene dynamics in the HSPC compartment during the induction of inflammation. Whereas global groups encompass diversity in gene patterns, cluster-enriched gene groups show far less variation and more specificity, with HSCs being the fast responders and recovers, whereas committed myeloid progenitors showing sustained down-regulation of genes.

### Single-cell abundance analysis shows myeloid depletion and HSC enrichment after IFNα treatment

**Figure 5.**
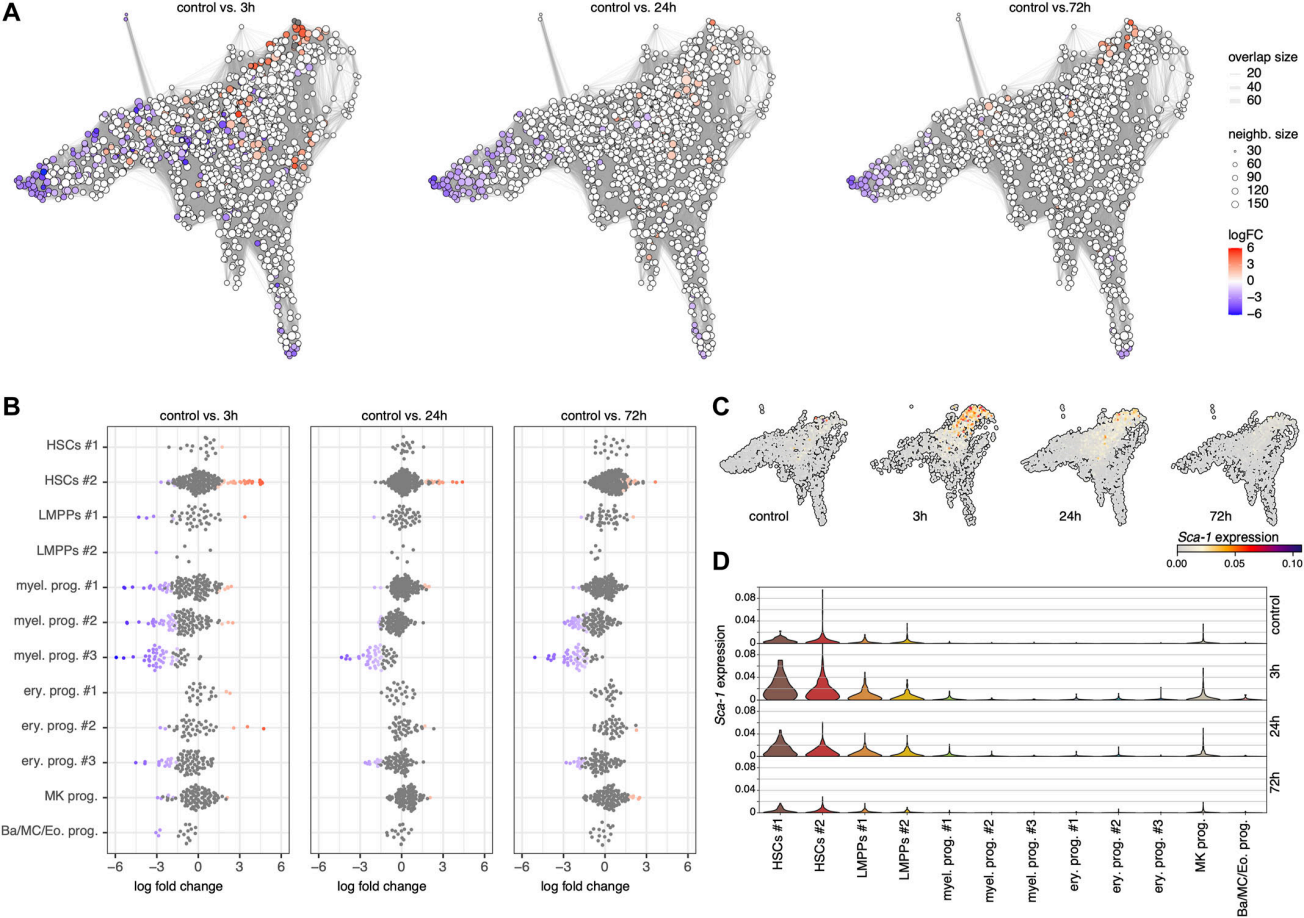
Abundance analysis reveals a sustained reduction in myeloid progenitors after IFN⍺ treatment. **(A)** Neighborhood graphs with the results from Milo differential abundance testing between the control dataset and post IFNα treatment subsets (3, 24, and 72 h). Nodes represent neighborhoods, coloured by their log fold change (red: more abundant, blue: less abundant, white: non-differentially abundant). Graph edges represent the number of cells shared between two neighborhoods. **(B)** Beeswarm plot of the distribution of log fold changes in each cluster. Neighborhoods are assigned to clusters based on the most commonly found cluster label in the neighborhood. **(C, D)** UMAP embeddings (C) and violin plot (D) of *Sca-1* expression in the control, 3, 24, and 72 h timepoints.

**Figure S6.**
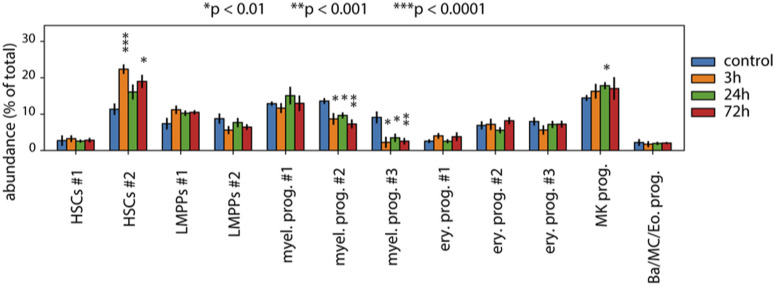
Relative abundance decreases in myeloid progenitors. Relative abundance of clusters in each time point. Statistical significance was determined by a two-sided *t* test between the relative abundance of a cluster in control and each treatment time point.

To investigate whether reduced transcriptional programs for myeloid differentiation and function upon IFN⍺ treatment also impacted the size of the progenitor compartment, we performed a differential abundance analysis on the level of clusters and neighborhoods of cells. We applied the Milo algorithm (Dann et al, 2022) which models cellular states as overlapping neighborhoods on a KNN graph rather than relying on clustering cells into discrete groups (see the Materials and Methods section). At a false discovery rate of 10%, we could observe multiple neighborhoods that were differentially abundant (Fig 5A). Neighborhoods received a cell-type label based on the most predominant cluster in the neighborhood. Even though most progenitor-enriched clusters showed a reduction at 3 h, most returned to normal by 24 or 72 h, except for the most differentiated myeloid progenitors (Myel. prog. #2 and Myel. prog. #3), which were significantly reduced, even at 72 h posttreatment (Figs 5B and S6). The abundance of HSCs increased in HSCs #2 posttreatment, most significantly at 3 h (Figs 5B and S6). This can be explained by our strategy of FACS enrichment of rare cell types (the Materials and Methods section) and the fact that the total number of cells captured at the 3-h time point was smaller (the Materials and Methods section) compared with the other time points, giving the enriched HSC population a higher abundance in the 3-h sample. Our abundance analysis in the HSPC compartment also showed that acute IFN⍺ treatment resulted in a sustained significant reduction in the most committed myeloid progenitors over the whole time course of the response. This is in contrast to the current notion in the field claiming that the decreased frequency of LS^−^K (comprising myeloid, erythroid, and megakaryocytic progenitors) and concurrent increase in LSKs (comprising HSCs and LMPPs) upon IFNα stimulation (Fig 1B–E) is mainly the result of contaminating myeloid progenitors that have reacquired *Sca-1* expression (and would fall into the LSK gate) (Pietras et al, 2014; Kanayama et al, 2020). However, when analyzing *Sca-1* gene expression in our dataset, the absolute gene expression of *Sca-1* in the myeloid progenitors never exceeds the gene expression levels of the HSCs, even though there is a relative change in each of the clusters (Fig 5C and D). Thus, this unbiased (i.e., transfer of cell type labels from the control, based on the expression of several genes) investigation of the different clusters and their abundances identifies a true change in myeloid population size and not a shift in populations because of marker change in response to inflammation.

### Myeloid depletion coincides with changes in transcriptional programs

**Figure S7.**
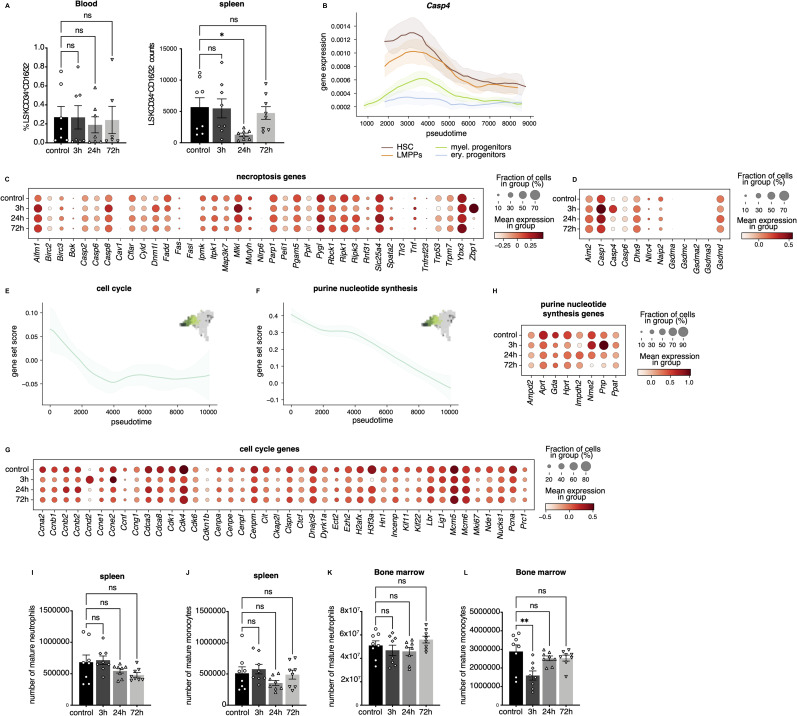
Reduction of myeloid progenitor functional properties after IFN⍺ treatment. **(A)** Flow cytometry analysis of of myeloid progenitors (Lin^−^ Sca-1^−^ cKit^+^ CD34^+^ CD16/32^+^) in WT mice at 3, 24, and 72 h after IFN⍺ or control (PBS) treatment in blood (left; frequency) and spleen (right; counts), respectively. n = 8 biological replicates. **(B)** Pseudotemporal expression of pyroptosis genes *Casp4* in pooled HSCs, LMPPs, myeloid and erythroid progenitors. **(C, D)** Expression of necroptosis (C) and pyroptosis (D) associated genes in myeloid progenitors in the four experimental subsets. **(E, F)** Score of cell cycle (E) and purine nucleotide synthesis (F) genes in the pooled myeloid progenitor clusters plotted in pseudotime. **(G, H)** Expression of cell cycle (G) and purine nucleotide synthesis (H) associated genes in myeloid progenitors in the four experimental subsets. **(I, J)** Flow cytometry analysis of counts of neutrophils (B220^−^ CD4^−^ CD8^−^ Ly6G^+^ CD11b^+^) (I) and monocytes (B220^−^ CD4^−^ CD8^−^ Ly6G^−^ CD11b^+^ CD11c^−^ F4/80^−^) (J) in spleen. **(K, L)** Flow cytometric analysis of counts of neutrophils (B220^−^ CD4^−^ CD8^−^ Ly6G^+^ CD11b^+^) (K) and monocytes (B220^−^ CD4^−^ CD8^−^ Ly6G^−^ CD11b^+^ CD11c^−^ F4/80^−^) (L) in bone marrow. Statistical significance in (A, I, J, K, L) was determined by an ordinary one-way ANOVA using Holm–Šídák’s multiple comparisons test and at least two independent experiments were performed; *P ≤ 0.05, ***P ≤ 0.001, ****P < 0.0001.

**Figure 6.**
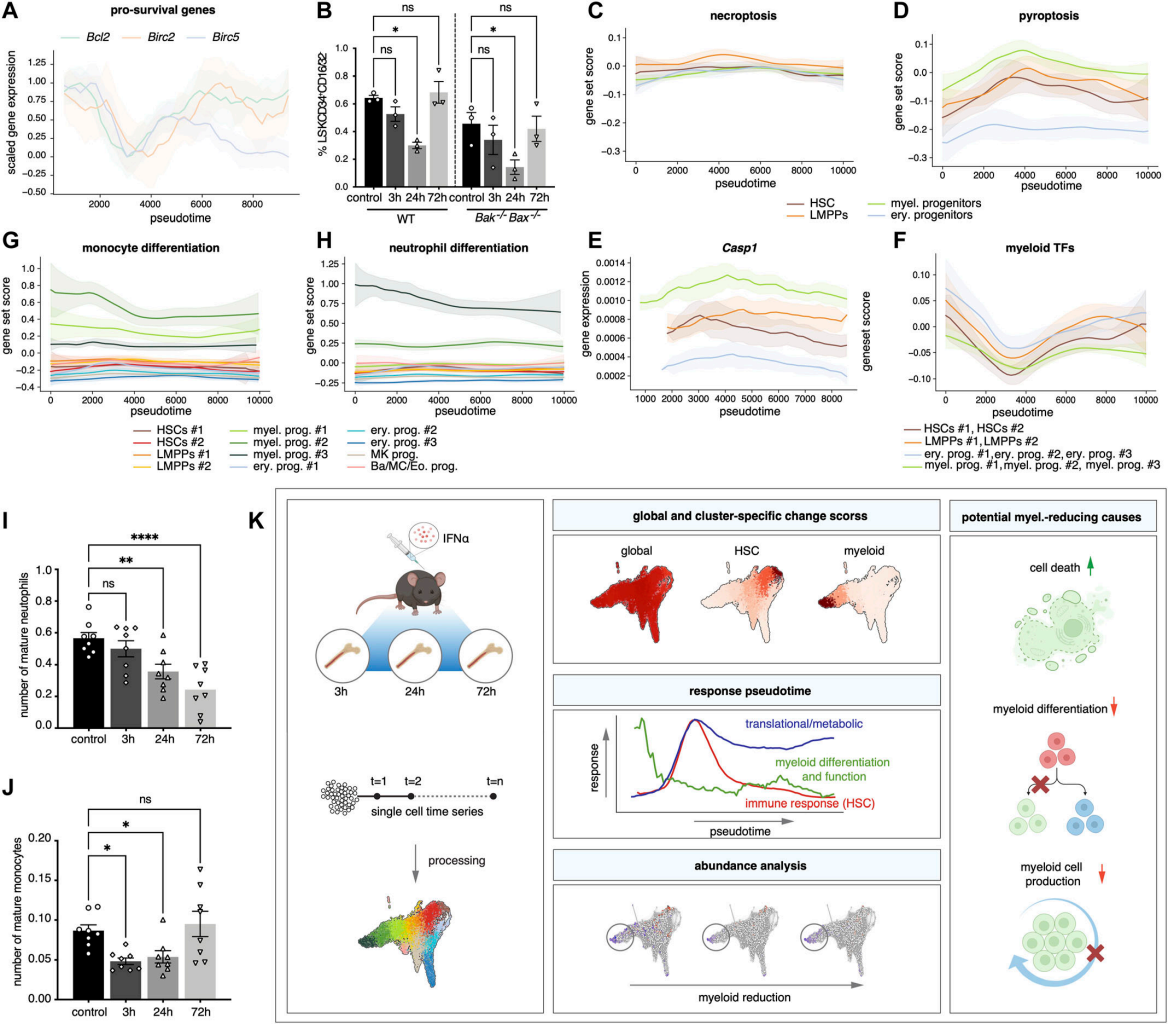
Reduced myeloid differentiation bias and increased cell death signature partially explain myeloid population’s reduction upon inflammation. **(A)** Pseudotemporal expression of pro-survival genes (*Bcl2*, *Birc2*, *Birc5*). **(B)** Flow cytometric analysis of BM frequency of myeloid progenitors (Lin^−^ Sca-1^−^ cKit^+^ CD34^+^ CD16/32^+^) after IFN⍺ treatment in WT and *Bax*^*−/−*^*Bak*^*−/−*^ double knockout mice at 3, 24, and 72 h after IFN⍺ or control (PBS) treatment. n = 3 biological replicates. **(C, D)** Score of necroptosis gene signature (C) and pyroptosis gene signature (D) in pooled HSCs, LMPPs, myeloid and erythroid progenitors plotted in pseudotime. **(E)** Pseudotemporal expression of pyroptosis genes *Casp1* in pooled HSCs, LMPPs, myeloid and erythroid progenitors. **(F)** Score of (murine) myeloid transcription factors in pooled HSCs, LMPPs, myeloid and erythroid progenitors plotted in pseudotime. **(G, H)** Score of monocyte (G) and neutrophil (H) differentiation genes in all clusters plotted in pseudotime. **(I, J)** Flow cytometric analysis of blood neutrophils (B220^−^ CD4^−^ CD8^−^ Ly6G^+^ CD11b^+^) (I) and monocytes (B220^−^ CD4^−^ CD8^−^ Ly6G^−^ CD11b^+^ CD11c^−^ F4/80^−^) (J) normalized to the whole blood leukocyte count as measured by hemavet at 3, 24, and 72 h injection of IFNα or control (PBS) treatment in WT mice. n = 8 biological replicates. **(K)** Graphical abstract of the article. **(I, J)** Statistical significance in (I, J) was determined by an ordinary one-way ANOVA using Holm–Šídák’s multiple comparisons test and at least two independent experiments were performed; *P ≤ 0.05,**P ≤ 0.01, ***P ≤ 0.001, ****P < 0.0001. Data represent mean ± SEM.

The reduction in the abundance of the myeloid progenitors could be caused by an increased egress of these cells from the bone marrow, a loss of these cells because of cell death, or a reduction in differentiation towards myeloid progenitors. Myeloid progenitors were not observed in the blood at any time point after IFNα treatment (Fig S7A). However, the number of myeloid progenitors in the spleen decreased at 24 h (Fig S7A), in line with the reduction observed in the bone marrow (Fig 5A and B). This suggests that the reduced abundance of the myeloid progenitors in the bone marrow is not because of increased egress into the blood or spleen. To investigate whether reduced levels of myeloid progenitors in the bone marrow were the result of increased cell death, gene patterns of pro-survival genes (*Bcl2*, *Birc2*, and *Birc5*) were analyzed and found to be decreased after IFNα treatment (Fig 6A). Yet, in *Bax*^*−/−*^*Bak*^*−/−*^ double-knockout mice, in which cells are unable to undergo apoptosis because of the loss of the pro-apoptotic proteins *Bax* and *Bak*, a similar reduction of myeloid progenitors was observed in the bone marrow as in wild-type mice (Fig 6B), indicating that apoptosis was not the reason for the reduction in myeloid progenitors. This does not exclude the involvement of other forms of cell death, like necroptosis and pyroptosis. Expression of necroptosis-related genes (Fig 6C) was not significantly altered in any of the cell types; however, expression of some pyroptosis-related genes (Fig 6D) such as *Casp1* and *Casp4* (Figs 6E and S7B–D), was increased in the myeloid progenitor subpopulations. Together, these data suggest an early increase in pyroptosis rate in the myeloid progenitors compared with the other cell types in response to the treatment, possibly resulting in a reduced abundance of these cells.

To investigate whether insufficient production of new cells might also play a role in the sustained reduction in myeloid progenitors, the expression of genes involved in myeloid lineage priming was analyzed. By calculating a gene score for known myeloid transcription factors that have been reported to impact both stem and progenitor cells, a global down-regulation in the myeloid program (including priming in HSCs and the more differentiated LMPPs) was present in the early stages of the IFNα response (Fig 6F). In addition, a reduction in cell cycle and purine nucleotide synthesis genes was observed in myeloid progenitors, suggesting that both myeloid differentiation in HSCs and LMPPs and cell production in myeloid progenitors, could be affected (Fig S7E–H).

Along with the reduction in myeloid differentiation related genes, neutrophil, and monocyte transcriptional signatures were also down-regulated in myeloid progenitors over the pseudotime axis (neutrophil: myel. prog. #1; monocyte: myel. prog. #3) (Fig 6G and H), suggesting continuously reduced differentiation of myeloid progenitors to mature myeloid cells. This was confirmed by a gradual decrease in the number of neutrophils in the blood over time (Fig 6I). The number of monocytes in the blood also decreased in response to treatment, but already recovered at 72 h (Fig 6J). Similar trends were observed in bone marrow and spleen; however, no significant changes were detected (Fig S7I–L). Taken together, these in vivo cell analyses and gene expression data indicate an increase in pyroptosis signature combined with reduced myeloid differentiation both at the stem cell level, and in committed progenitors, accompanied with a reduction in the cell production machinery in progenitors, resulting in reduced myeloid progenitors in the bone marrow, and lower levels of mature myeloid cells in the blood (Fig 6K).

## Discussion

Analysis of single-cell RNA-seq time series is nontrivial because of its high complexity, regarding the inclusion of multiple cell types, a high number of genes, and the extra dimension of (pseudo-) time. However, these types of experiments allow for marker-independent, unbiased analysis of dynamic responses of heterogeneous cell populations, such as the response of the HSPC compartment to inflammatory stress. To overcome the challenges of analyzing such datasets, we designed a computational pipeline that bundles application of our novel approaches and established computational tools (e.g., Scanorama, DEseq 2, Milo) for processing and analyzing single-cell RNA-seq time series. Our pipeline labels clusters based on the expression of multiple (n = 3,326) genes after correcting for treatment effects (Fig 6K), thus avoiding relabeling of the cells undergoing inflammation as separate populations. This will ensure the study of the same cell type over time. Hence, cell-type identity is reliably retrieved, even though the conventional marker genes might be subject to changes, as is the case during inflammation.

Furthermore, we designed measures that make the temporal dynamics more comprehensible and provide a (visual) entry point into all the information in the data. Unsupervised methods that are merely based on cell-to-cell proximities are unable to capture the pseudotemporal order of the cells in our data, where the cell states after relaxation become more similar (but not completely indistinguishable) to the cell states before treatment. Therefore, we implemented a semi-supervised method, that is, using the experimental time label information, for inference of response pseudotime. Using a minimal linear regression model, our response pseudotime reconstruction enabled capturing the fine-grained expression changes and dynamical patterns beyond the four discrete experimental time points (Fig 6K). Recently, alternative semi-supervised methods such as psupertime (Macnair et al, 2022) or approaches suitable for temporal patterns that a linear matrix transformations cannot capture (e.g., periodic dynamics or other cases of complete indistinguishability of expression between two time points) are being investigated. Overall, our methods and pipeline can be used by researchers studying a posttreatment (response) process using a single-cell time series.

Although many studies have investigated the role of inflammation on HSC function, changes in marker expression on these cells have made it challenging to examine the impact of inflammation on the heterogeneity and molecular changes over time in the HSPC compartment. Our unbiased cell type annotation approach and response pseudotime analysis allowed us to highlight the dynamic nature of HSPCs’ response to IFNα with global and cell-type–specific distinct molecular patterns of gene expression and biological processes (Fig 6K). Whereas global gene expression groups and patterns were heterogeneous in dynamics and linked to diverse processes such as metabolism, translation, and inflammation, HSC-specific gene groups and patterns followed rapid sensing, response, and recovery and were enriched for distinct inflammation-related genes, suggesting global and stem cell-specific responses to inflammation. Our response pseudotime analysis shows that most gene expression patterns reach a steady-state plateau within 72 h (Fig 3C), implying that we are capturing most dynamical expression changes of the system upon IFN-α treatment. However, the few patterns that do not reach such a plateau (pattern 5, 8, and 12) indicate that there are ongoing dynamical changes which extend beyond the time frame studied here and potential development of further cascades of molecular changes. Moreover, many gene patterns (patterns 1, 2, 3, 11, 12, etc.) reached a plateau which is higher or lower than their before-treatment value. This indicates irreversible changes that leave a mark in the activated cells (Bogeska et al, 2022). Thus, our data show that a single inflammation cytokine treatment may already include irreversible components, something that needs further investigation by extending the time series analysis to later time points up to 1 wk or even longer.

Emergency myelopoiesis, that is, increased production of myeloid cells, has been described in response to many pro-inflammatory cytokines and infections (Manz & Boettcher, 2014). However, thus far, we have not been able to identify any impact on myeloid production or differentiation upon IFNα treatment because of extensive changes in stem cell-specific marker expression upon inflammation (Demerdash et al, 2021). Others claimed that decreased frequency of myeloid progenitors and the simultaneous increase in LSKs upon in vitro treatment of HSPCs with IFNα was mainly the result of myeloid progenitors reacquiring Sca-1 expression (Pietras et al, 2014; Kanayama et al, 2020). With our unbiased investigation of the different clusters, defined solely by their gene expression, we could now show that IFNα-induced *Sca-1* gene expression only occurred in immature HSCs and LMPPs (Fig 5C and D). Even though CITEseq analysis of HSPCs should be performed to confirm these results at the Sca-1 protein level, our data do indicate that the LSK expansion observed in flow cytometry is mainly because of the enrichment of the HSCs and multipotent progenitors and not myeloid progenitor populations shifting into the LSK gate.

Unlike other pro-inflammatory cytokines such as TNFα (Yamashita & Passegué, 2019) and IL1β (Pietras et al, 2016), we did not find characteristics typical of emergency myelopoiesis upon IFNα treatment. Instead, abundance analysis showed a decrease in myeloid progenitor numbers; gene expression related to myeloid priming was down-regulated in all clusters from immature HSCs to committed myeloid progenitors; and myeloid-derived mature neutrophils were continuously reduced in the blood (Fig 6K). In addition, response pseudotime analysis revealed changes in the expression of genes related to pyroptosis, suggesting that reduced levels of myeloid progenitors might be the result of a combination of impaired myeloid differentiation with increased myeloids cell death rate via pyroptosis. Interestingly, upon infection with *Mycobacterium tuberculosis*, HSCs are reprogrammed to limit their commitment towards myelopoiesis via a type I IFN-signaling axis (Khan et al, 2020). In this same study, they showed that IFNα induces RIPK3-mediated necroptosis in myeloid progenitors. However, RIPK3 is a component of both pyroptosis and necroptosis depending on other proteins participating in these pathways (Shlomovitz et al, 2017). Differentiation and cell death pathways are not only regulated at the transcriptional level. Thus posttranslational analysis and additional functional approaches need to be performed to unravel further the programs controlling the IFNα-induced reduction in the myeloid progenitors in the bone marrow. Thus, our time course data suggest an unanticipated impact of IFNα on the differentiation, production, and death of myeloid cells, highlighting the diverse impact of the same pro-inflammatory agonist on related but distinct cell types at different time points in the response. This link between IFNα and reduced production and levels of myeloid cells such as neutrophils not only helps us to better understand the impact of inflammation on the whole hematopoietic compartment, but will also help to understand better the role of IFNα in disease settings such as the autoimmune disease systemic lupus erythematosus in which neutrophil dysfunction plays an integral role in disease pathogenesis (Kaplan, 2011) and IFNα is associated with adverse outcomes (Rönnblom & Leonard, 2019).

Altogether, our pipeline designed for posttreatment single-cell time series data has shed light on the ways in which different cell types, genes, and processes in the HSPC compartment are modulated during the different phases of acute, IFNα-induced, inflammation response.

### Limitations of the study

By creating this resource, we have established a foundation for exploring the functional properties of the molecular signatures of HSPCs under inflammatory stress. Whereas correlations inferred from expression data offer hypotheses for regulatory mechanisms, experimental testing is crucial for confirming these hypotheses. However, inflammation-induced marker changes on HSPCs are still a limitation to perform cell cluster-specific functional follow-up studies. Recently developed CITEseq analysis would allow us to identify better markers to distinguish the different stem cell and progenitor populations under inflammation to allow better isolation of these populations for future functional analysis. Moreover, additional layers of regulation at the chromatin and protein level impact and control cell behavior, which may manifest as unappreciated heterogeneity and dynamic properties. Integrating our time series data with methylation, chromatin accessibility or proteome analysis will achieve further comprehensive modeling of the regulatory and signaling networks coordinating the stress response.

We acknowledge the possibility of changes in cell abundance as a result of our rare HSC population enrichment. However, we emphasize that the significant decrease in myeloid progenitors persisted consistently across all time points, indicating a true biological change rather than an artifact (Figs 5A and B and S6). Whereas we recognize the potential influence of our enrichment for HSCs on cell abundance of other populations, the abundance reduction was specific to the myeloid population rather than a uniform reduction across all populations (as would be expected in compensation for HSC numbers). We further validated the reduction of myeloid progenitors in both blood and spleen (Fig S7); this further confirms our HSPC single-cell abundance results.

Our combined abundance and response pseudotime analysis suggest a link between myeloid progenitor reduction in the bone marrow and the HSCs reduced myeloid differentiation bias and a potential enhancement of cell death mechanisms in the myeloid progenitors, relative to the other HSPC populations.

Validation of these findings at the transcriptional level is however not straightforward because of the multitude of posttranscriptional regulatory mechanisms involved until a specific biological function (e.g., enhanced cell death) is manifested in the system. We also sought to check if the post-inflammation decline in differentiation bias towards myeloid progenitors could be confirmed by a comparison of RNA-velocity of the cells at the different time points. We estimated RNA-velocities of the cells in the control data set (Marot-Lassauzaie et al, 2022). However, meaningful comparison with the cell velocities of later time point datasets was hindered by the yet insufficient robustness of state-of-the-art velocity parameter inference and the poor quantification of unspliced versus spliced mRNA, especially when using the 3′-end gene expression short reads captured by the 10X sequencing platform (Marot-Lassauzaie et al, 2022). Emergence of more reliable approaches for estimating cell velocities could be a valuable asset as an orthogonal dynamical analysis approach to time series and pseudotime analysis, before we (or other researchers) turn to perform further costly and challenging functional and experimental validations in the future.

## Materials and Methods

### Mouse models

All animal experiments were approved by the local Animal Care and Use Committees of the German Regierungspräsidium Karlsruhe für Tierschutz und Arzneimittelüberwachung. Mice were kept under specific pathogen-free conditions in ventilated cages (ICV) in the animal facility of the German Cancer Research Center (DKFZ). Mice used for experiments were between 10–20 wk old at the beginning of the respective experiments. *SclCreERT bax*^*−/−*^*bak*^*−/−*^ mice were on a C57BI/6 background (Takeuchi et al, 2005), and treated for 5 d with 2 mg/d tamoxifen. IFNα treatment was started 4 wk post tamoxifen treatment. C57Bl/6 (WT) mice were bred at the DKFZ animal facility or bought from JANIVER lab. Mice were euthanized by cervical dislocation according to German guidelines.

### IFNα treatment of mice

Mice were injected subcutaneously with 50,000 international units (IU) of recombinant mouse IFNα per 20*g* mouse (Milteny Biotech). Recombinant mouse IFNα was diluted in PBS and control mice were injected with 100 μl PBS.

### Isolation of BM, spleen, and blood for flow cytometry analysis

Blood was collected from the vena facialis by sub-mandibular bleeding into EDTA-coated collection tubes. Blood was either analyzed automatically with a Hemavet cell counter (Drew Scientific) or stained for flow cytometry after initial RBC lysis by incubation with ACK lysis buffer for 20 min. Cells were stained for Ter119, CD4, CD8, CD11b, CD11c, Ly6G, B220, F4/80. BM cells were isolated from the femur, tibia, hip bone, and spine by bone-crushing. Splenocytes single-cell suspension was obtained by mashing the spleen through a 40 μm EASYstrainer (greiner bio-one). After ACK, lysis BM cells and splenocytes were stained using antibodies for CD117 (cKit), Sca-1, CD150, CD48, CD34, CD16/32, and lineage antibodies (CD4, CD8, CD11b, Gr-1, B220, and Ter119). For the BrdU incorporation assay, BrdU (18 mg/kg; Sigma-Aldrich) was administered i.p. for 14 h before harvesting the BM. The BD Pharmingen BrdU Flow Kit protocol was used to stain for BrdU. For flow cytometry analysis the LSR Fortessa or LSRII were used (BD Biosciences). Flow data were analyzed using BD FACS DIVA v8.0.1 and Flowjo (v10).

### FACS sorting

For FACS sorting of single cells, BM cells were isolated and RBC lysed as described above. This was followed by lineage depletion using a lineage antibody cocktail against CD4, CD8, CD11b, B220, Gr-1, and Ter119 and incubation with Dynabeads Magnetic Beads (Invitrogen). Lineage-depleted BM cells were stained with Zombie Yellow viability dye (BioLegend) followed by incubation with the following antibodies: CD117, Sca1, CD150, CD48, CD34, and lineage antibodies (B220, CD4, CD8, Ter119, Ly6G, CD11b, CD11c, and F4/80) together with one of the hash antibodies (TotalSeq-A0301 anti-mouse Hashtag 1 Antibody, TotalSeq-A0302 anti-mouse Hashtag 2 Antibody, TotalSeq-A0303 anti-mouse Hashtag 3 Antibody, TotalSeq-A0304 anti-mouse Hashtag 4 Antibody) (TotalseqA antibodies; BioLegend). The four biological replicates of each time point were stained with one of the four unique hash antibodies. Cells were sorted using a FACSAria Fusion or FACSAria II equipped with a 100 μm nozzle (BD Biosciences).

### Single-cell RNA library preparation and sequencing

HSPC single-cell RNA-seq was performed using the 10X Genomics platform. The Chromium Next GEM single cell 3′ reagent kits v3.1 were implemented to prepare the libraries, following the official instruction manual (https://www.10xgenomics.com/support/single-cell-gene-expression/documentation/steps/library-prep/chromium-single-cell-3-reagent-kits-user-guide-v-3-1-chemistry). Briefly, 10,000 Lin^−^ cKit^+^ cells were sorted and enriched for HSCs by sorting additional 3,000–4,000 Lin^−^ cKit^+^ CD150^+^ CD48^−^ CD34^−^ cells. Cells were super-loaded according to the manufacturer’s instructions up until the cDNA amplification step. 1 μl/sample of HTO primers was spiked into the cDNA amplification PCR, and cDNA was amplified according to the 10x Single Cell 3′ v3.1 protocol aiming for a targeted cell recovery of 500–6,000 cells. After PCR, cDNA cleanup was performed by using SPRI to separate the HTO-derived cDNAs (in the supernatant) from the mRNA-derived cDNAs (retained on beads). The cDNA fraction was processed according to the manufacturer’s protocol to generate the transcriptome library. The quality of the obtained cDNA library upon adapter ligation and sample index PCR was assessed on an Agilent Bioanalyzer High sensitivity chip. Library sequencing was performed on the Novaseq 6000 Illumina sequencing platform.

### Filtering longitudinal single-cell RNAseq dataset

The cellranger pipeline (version 3.1.0) was used to align all reads to the mm10 genome and count the coverage of each gene in each cell. Based on the hashtag barcodes, cells were assigned to their corresponding time point (control, 3, 24, or 72 h) and batch (four batches per time point). Cells with multiple barcodes (multiplets) or missing barcodes (negatives) were removed from the dataset. In the resulting count matrix (cells × genes), cells with a high amount of mitochondrial genes (>5%) or a low amount of unique genes (<700) were filtered out. After the filtering steps, the following number of cells was present in each of the respective time points: control—2,474, 3 h—1,661, 24 h—3,462, 72 h—2,449 (10,046 cells in total).

### Clustering and cell type annotation

The 500 most highly variable genes (HVGs) were identified in the control subset using analytic Pearson residuals (Lause et al, 2021). The control subset was subsetted for the 500 HVGs, and the counts were L2 normalized. Next, a neighborhood graph was computed using 10 out of 50 principal components and the 15 nearest neighbors. The Leiden algorithm (resolution = 0.8) identified 14 distinct clusters in the control subset (Traag et al, 2019). Each cluster was appointed to a cell type based on (1) DEGs between the cluster of interest and all other clusters, (2) the expression profiles of the HVGs, (3) known marker genes, and (4) correlation with cell types in a previously published dataset of the HSPCs (Nestorowa et al, 2016).

### Label transfer and UMAP representation

We identified the top 2,000 HVGs in each subset and subsetted the complete dataset with the combined list of HVGs. Afterward, the dataset was L2 normalized, and the different subsets were integrated using Scanorama (Hie et al, 2019). All 100 Scanorama-reduced dimensions were used to calculate a neighborhood graph (nearest neighbors = 15). A two-dimensional UMAP representation was computed using the neighborhood graph. To transfer the cell-type labels from the control subset to the response subsets (3, 24, and 72 h), cells in the response subsets would adopt the cell type label that was most common among their 15 nearest neighbors (Euclidean distance) in the control subset. The integrated data were only used for label transfer and visualization purposes. For other downstream analyses, we use the filtered-only dataset. In this dataset, we removed the Ba/MC/eos. cells and monocytes because of the small number of cells assigned to those cell types (10 and 52, respectively).

### Calculating gene set scores

The filtered dataset was L2 normalized and scaled to unit variance and zero mean. The ISG score was calculated by subtracting the average expression of a random set of reference genes from the average expression of about 400 known ISGs (Scanpy function score_genes). Similarly, the stemness (Giladi et al, 2018), necroptosis (GO:0070266), pyroptosis (GO:0070269), myeloid TF (Kwok et al, 2020), monocyte and neutrophil differentiation, cell cycle (Giladi et al, 2018), and purine nucleotide synthesis (Vogel et al, 2019) score were calculated. Genes for each signature are available as a .csv file. Necroptosis and pyroptosis gene sets were retrieved from the Mouse Genome Database, Mouse Genome Informatics, The Jackson Laboratory. World Wide Web (URL: http://www.informatics.jax.org). (The data were retrieved in the year 2022).

### Differential abundance analysis

We used the R package Milo to perform an abundance analysis on the L2 normalized, filtered dataset (Dann et al, 2022). A neighborhood graph was built using 30 out of 100 of the Scanorama-reduced dimensions (see *Label transfer and UMAP representation*) and 30 nearest neighbors. Afterward, we followed the steps described in the accompanying tutorial (*Milo example on mouse gastrulation dataset*) for each response subset (3, 24, and 72). In each analysis, the control subset served as the reference, to which the response subset would be compared.

### Identifying response genes

We used the edgeR-LRT method in the Libra R package to find the DEGs between the control and any of the response subsets, in each cluster. We considered only DEGs with an adjusted *P*-value higher than 0.05 and a log-fold change higher than 1 in at least one cluster. For the downstream analyses of the response genes, we consider only the 500 DEGs with the highest significance. In case a DEG is found in more than one cluster and/or time point, we take only the lowest *P*-value into consideration.

### Change score

We L2 normalized the filtered dataset per cell. For each response gene (*i*) we took the mean expression (μ) in each cluster (*j*) per time point (*t*). The expression change was calculated as the absolutes sum of the derivative of the mean expression across all time points (here *m* = 3 for control-3, 3–24, 24–72 h). Thus the change score (*c*_*i*,*j*_) per cluster for each response gene:

$$c_{i,j}=\overset{m}{\underset{t=1}{\sum }}\left|\mu_{i,j,t+1}-\mu_{i,j,t}\;\right|$$ (1)

The result is a matrix with change scores per cluster for each of the response genes. We applied hierarchical clustering and grouped the response genes into 14 groups by setting a threshold at the cophenetic distance of 3 (Scipy function cluster.hierarchy.linkage and cluster.hierarchy.fcluster).

### Similarity score

We define the similarity score between the cluster-specific expression profiles of gene *i* and the expression profile of the same gene in the complete dataset in two steps. First, both the expression of the cluster and the complete dataset were scaled between 0 and 1 by min–max normalization (with *n* = 4 indicating the number of time points in the time series).

$${\underline{x}}_{i,j,t}=\frac{\mu_{i,j,t}\;-\;min_{t}\;\mu_{i,j,t}}{max_{t}\;\mu_{i,j,t}\;-\;min_{t}\;\mu_{i,j,t}}$$ (2)

Note that *max*_*t*_ *μ*_*i*,*j*,*t*_ and *min*_*t*_ *μ*_*i*,*j*,*t*_ indicate the max/min of the mean expression for gene *i* cluster *j* among all time points *t*. Second, we calculate a dissimilarity score between the cluster and the whole data set for each response gene by subtracting the cluster-specific expression changes from the expression changes in the complete dataset. Third, the magnitude of these differences were summed and normalized by the number of time points. Finally, we turn the dissimilarity into a similarity score (*s*_*i*,*j*_) by subtracting it from 1 such that 1 presents complete similarity to the average behavior of all cells in the dataset.

$$s_{i,j}=1\;-\;\frac{\overset{n}{\underset{t=1}{\sum }}\left|{\underline{x}}_{i,j=all,t}\;-\;{\underline{x}}_{i,j,t}\right|}{n}$$ (3)

The result is a matrix with similarity scores per cluster for each of the response genes.

### Pseudotemporal ordering of cells during response

We opt to find a pseudotime axis that correlates with the actual arrow of time. Thus, we look for a transformation (*W* of size [*G*,1]) of the expression data from all time points that reconstructs the experimental time point of each cell with minimal error (ε):

X∗W=T+ε (4)

Here, *X* (of size [*N*,*G*]) is the filtered count matrix after per-cell L2 normalization, subsetting for all response genes and per-gene standardization (i.e., setting the mean to zero and variance to 1). The per cell L2 normalization is part of our single-cell data preprocessing routine (also see Haghverdi et al [2018]) on our preference of L2 normalization rather than size factor L1 normalization). The per-gene standardization is for correct calculation of the *X*^*T*^ *X* matrix (also necessary for other methods such as principal component analysis), such that the different features become comparable; for example, genes with overall high expression do not artificially seem highly correlated to other genes. Thus, by data standardization, we ensure mean- and variance-independent calculation of the weights in the W matrix. *T* is a vector (of size [*N*, 1]) with an (experimental) time assignment for each cell, created by taking the experimental time points (control, 3, 24, and 72 h) and converting those to 0, 1, 2, and 3 values respectively (as an alternative to such equal importance separation of the time points, one could consider using the actual time values on a log-scale). By this choice, we imply equal interest in separating each time point from the subsequent time point, whereas e.g., using the actual time values (0, 3, 24, and 72) would mean a larger emphasis on finding separation between the last two time points with the largest distance (i.e., 72−24 = 48).

In this work we have used the regression model (without an intercept) on standardized expression values and time scale choice as described above. Nevertheless, one could also consider other equally well justifiable variations, for example, a regression model including an intercept term. Note that the notion of pseudotime, which is generally based on a transformation of cell states’ profiles (e.g., gene expression), provides only a flexible estimation of the dynamical progression order of the cells and is not an absolute or unique quantity (e.g., when using slightly different transformations) as discussed in supplementary note 6 of Haghverdi et al (2016), and also demonstrated on simulation data (Fig S5J–M). The least squares analytical solution for *W* (which minimizes *ε*^*T*^ ∗ *ε*) is given by:

$$W={\left(X^{T}X\right)}^{-1}*X^{T}*T$$ (5)

We used the expression matrix *X* with the size of 10,046 cells and 2,501 genes and the cells’ corresponding time labels to solve the above linear regression problem. We note that, to avoid over-parametrization and to ensure the identifiability of the solution, the number of cells has to be larger than the number of genes. After *W* has been retrieved, a pseudotime coordinate can be calculated for each cell by the following:

PT=X ∗ W (6)

Where *PT* (of size [*N*, 1]) is the vector with the pseudotime coordinate for each cell. For further downstream analyses, the cells were ordered based on their pseudotemporal assignment. Note, the genes which the model forms a linear combining of, do not necessarily need to be linear or monotonic themselves. In fact, on simulation data, we show the model is able to reconstruct the pseudotime, (up to an acceptable correlation with the true time orders), even in a case where all genes are non-monotonic (complex) in the data (Fig S5I).

To cluster the response genes based on their pseudotemporal expression pattern, we smoothed the expression of these genes by taking the mean expression per 100 cells over the pseudotime axis. The expression values were scaled between 0 and 1 by min–max normalization. The 500 response genes were then clustered into 16 pseudotemporal expression patterns, using hierarchical clustering (threshold at cophenetic distance of 5.2).

### Simulation of time series

For the discrete time simulation, we sampled 1,000 cells from four different discrete timepoints: 0, 5, 10, and 20 (250 cells per timepoint). To model the asynchronous progression of time, we added a Gaussian noise (SD 1.5) to the discrete sampling time of each cell, giving us each cell’s hidden pseudotime *t*. For the continuous time simulation, we uniformly sampled cells pseudotime *t* between 0 and 20. The expression dynamics of 20 different genes were then simulated using either a single sigmoid function: $S\left(t|\alpha ,\beta \right)=\frac{1}{1+e\left(\left(-t-\alpha \right)/\beta \right)}$ with randomly sampled offset *α* and width *β* (monotonic genes) or a sigmoid function deactivated at a switching time point *t*_*switch*_ : *S*(*t*|*α*,*β*) - *S*(*t* - *t*_*switch*_|*α*,*β*) (complex genes). The genes are then min–max normalized and Gaussian noise SD of 0.05 is added to each cell’s expression to simulate biological variance (see also Fig S5).

### Gene expression over pseudotime

Expression profiles of individual genes in response pseudotime (such as Fig 4B) were derived using a combination of bin smoothing and bootstrapping. To find the expression profile in the complete dataset, bin smoothing with a 600-cell window size was performed on a sample of 50% of the cells in the dataset. This was repeated 20 times to find the mean expression, which defines the expression profile. The 95% confidence intervals were calculated by multiplying the standard error with 1.96 and subtracting or adding to the mean. For the cluster-specific expression profiles of individual genes, a 50-cell window size was chosen instead, because of the smaller number of cells in each cluster.

### Gene score in pseudotime

The gene set score profile in response pseudotime was calculated using a combination of locally weighted least squares regression (LOESS) smoothing and bootstrapping. For each cluster, LOESS smoothing with a first-order regression model was applied to 50% of the cells. This was repeated 30 times. The score profile was derived by taking the mean and 1.96 times the standard error for the 95% confidence intervals.

## Data Availability

The single-cell RNA-seq data were deposited in the Gene Expression Omnibus (GEO) under accession code GSE226824.

### Code availability

All scripts used in this study are available on Github: https://github.com/bjbouman/time_series_analysis.

## Supplementary Material

Reviewer comments
